# Subacute Sclerosing Panencephalitis in a Toddler: Changing Epidemiological Trends

**DOI:** 10.1155/2013/341462

**Published:** 2013-12-12

**Authors:** Roosy Aulakh, Abhimanyu Tiwari

**Affiliations:** ^1^Division of Pediatric Neurology, Department of Pediatrics, Government Medical College & Hospital, Chandigarh 160030, India; ^2^Department of Pediatrics, Government Medical College & Hospital, Chandigarh 160030, India

## Abstract

Subacute sclerosing panencephalitis (SSPE) is a devastating “slow virus” brain disease resulting from persistent measles virus infection of neurons. The age at presentation is usually 8 to 11 years with onset usually occurring 2–10 years after measles infection. We report a 2-and-half-year-old boy who presented with progressively increasing myoclonic jerks and subtle cognitive decline. He was diagnosed as a case of SSPE based on clinical features, typical electroencephalographic finding, and elevated cerebrospinal fluid/serum measles antibody titers. He had measles 4 months prior to onset of symptoms. This case along with review of recently published reports suggests progressively decreasing latency period between measles infection and onset of symptoms observed in cases with SSPE. Clinical implication would mean investigating for SSPE even in infants or toddlers with compatible clinical features and recent history of measles infection.

## 1. Introduction

Subacute sclerosing panencephalitis (SSPE) is a devastating “slow virus” brain disease resulting from persistent measles virus infection of neurons. Fortunately, its incidence has decreased in developed countries as a result of large vaccination campaigns; it is still one of the commonest causes of progressive myoclonia with cognitive decline in developing countries with incomplete measles immunization coverage [1–4].

The age at presentation is usually from 8 to 11 years [5, 6] with onset usually occurring 2–10 years after measles infection [7]. We report a 2-and-a-half-year-old boy who presented with progressively increasing myoclonic jerks and subtle cognitive decline. He was diagnosed as a case of SSPE based on clinical features, typical electroencephalographic finding, and elevated cerebrospinal fluid/serum measles antibody titers. He had measles 4 months prior to onset of symptoms. This case along with review of recently published reports suggests progressively decreasing latency period between measles infection and onset of symptoms observed in cases with SSPE. Clinical implication would mean investigating for SSPE even in infants or toddlers with compatible clinical features and recent history of measles infection.

## 2. Case Summary

A previously well, two-and-a-half-year-old boy born to consanguineous parents and symptomatic for the last seven months presented with progressively increasing myoclonic jerks along with cognitive decline. He developed ataxia over the course of his illness and was bedridden for the last ten days prior to admission. He had measles infection 11 months back and had not received measles vaccination. There was no history suggestive of maternal measles during pregnancy. Prior to this illness, child was developmentally normal. He had 2 elder siblings both of whom were normal.

On examination, the child interacted poorly with the examiner and was not oriented to place or time, though he recognized his mother. He demonstrated frequent myoclonic jerks and had rigidity with brisk reflexes and extensor plantars. No focal neurodeficit or cranial nerve palsy was evident. Hearing was grossly normal. Visual deterioration was evident as child had difficulty in fixating at objects. Fundus examination revealed bilateral optic atrophy. He had no organomegaly and the rest of the systemic examination was essentially normal. Provisional diagnosis of grey matter neurodegenerative disease was considered and child was investigated.

**Figure 1:**
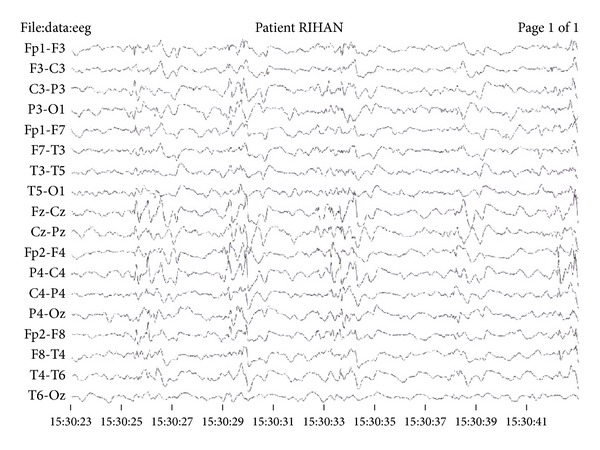
Twenty-second epoch of scalp electroencephalographic record revealing periodic bursts of high-amplitude, slow-wave complexes occurring every three to five seconds. Parameters are as follows: sensitivity 55 *μ*V/mm, time constant 0.3 s, and high-frequency filter 70 Hz.

**Figure 2:**
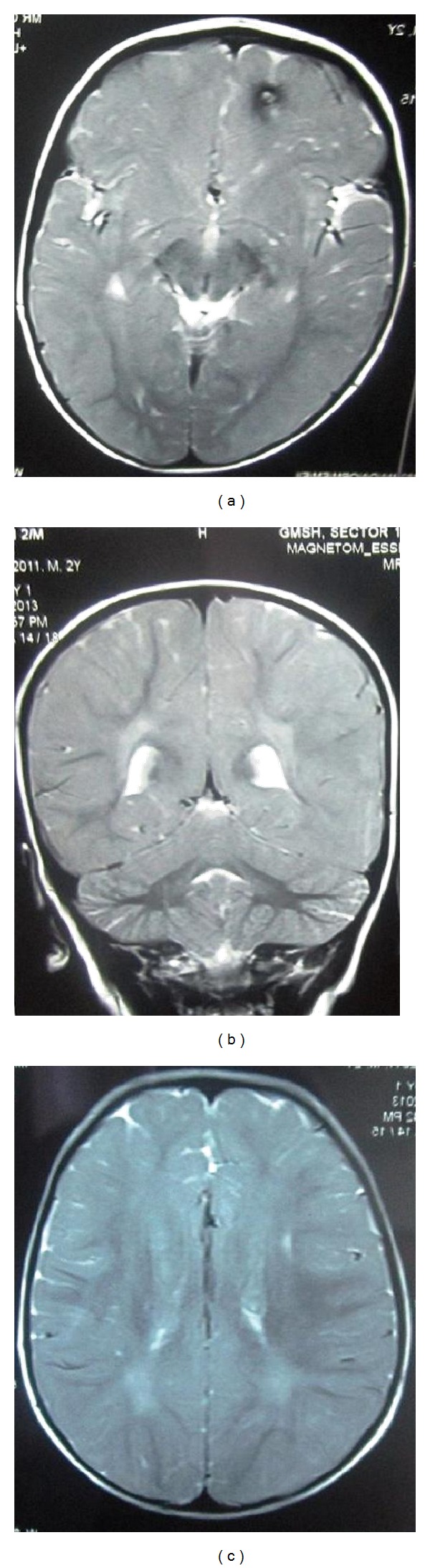
Magnetic resonance imaging of the brain. (a) T2 weighted axial section showing small hypointense focal lesion in left frontal lobe suggestive of cerebral contusion. (b) T2 weighted coronal section and (c) T2 weighted axial section showing ill-defined patchy areas of hyperintense signal in bilateral peritrigonal and paraventricular parietal white matter.

Routine hematological and biochemical parameters were all normal. Peripheral blood film did not reveal vacuolated lymphocytes. Scalp electroencephalogram revealed diffuse high amplitude bursts of periodic slow-wave complexes every three to five seconds, often accompanied by clinically evident axial myoclonus (Figure 1). Brain magnetic resonance imaging showed small focal lesion appearing hypointense on T2 weighted images, slightly hyperintense on T1 weighted images, and showing blooming on susceptibility weighted imaging seen in left frontal lobe suggestive of small cerebral contusion. In addition, ill-defined patchy areas of hyperintense signal on fluid attenuated inversion recovery magnetic resonance/T2 weighted images were seen in bilateral peritrigonal and paraventricular parietal white matter (Figure 2). Visual evoked potentials revealed bilaterally decreased amplitude. Blood tests for toxoplasma, cytomegalovirus, and herpes simplex virus IgG and IgM were negative. Cerebrospinal fluid (CSF) was acellular; CSF protein 34 mg/dL, CSF sugar 56 mg/dL against blood sugar of 102 mg/dL and measles antibody titers by enzyme immunoassay were raised in both cerebrospinal fluid and serum: 1 : 625 (positive > 1 : 4) and 1 : 625 (positive > 1 : 256), respectively, confirming diagnosis of SSPE. He tested negative for human immunodeficiency virus. Child was started on sodium valproate. Frequency of myoclonic spasms reduced significantly; however, therapy for SSPE could not be initiated because of financial restrains. Prognosis was explained to parents who decided to discontinue further therapy and took the child home.

## 3. Discussion

It is widely accepted that SSPE cannot occur in the absence of direct measles virus infection [8, 9].

The age at presentation is usually from 8 to 11 years [5, 6] with onset usually occurring 2–10 years after measles infection [7]. Measles continues to be a major cause of childhood morbidity and mortality in India. Recent studies estimate that 80,000 Indian children die each year due to measles and its complications, amounting to 4% of under-5 deaths. Hence, SSPE continues to be one of the commonest causes of progressive myoclonia with cognitive decline in developing countries with incomplete measles immunization coverage [1–4].

In this child, onset of symptoms of SSPE was very early, that is, at age of 23 months and latency period between measles infection and onset of symptoms was only 4 months. This trend of shorter latency period as observed in our child has recently been reported by a handful of researchers [10–15]. Various risk factors reported to influence the risk of chronic brain infection with the mutant measles virus include younger age at measles onset, living in a rural area, poverty, overcrowding, low level of parental education, an older mother, a higher number of siblings, and a higher birth order (i.e., elder sibling who would have higher chance of being exposed younger siblings with measles before the age of 5 years) [16]. Recently an association between programmed cell death protein 1 (PD-1), a member of the CD28 family, and children with SSPE has been reported [17]. Individuals with acquired immunodeficiency syndrome or children whose mothers have acquired immunodeficiency syndrome might be at higher risk of a fulminant course of SSPE [18], and earlier onset of familial cases of SSPE also has been reported to present with shorter latency period [19].

Few of these risk factors were operating in this case like poor socioeconomic status and overcrowding. Neurological deterioration in SSPE following head injury has also been reported in the literature [20]. This child did have history of head trauma at the age of 18 months and his brain magnetic resonance imaging revealed a small cerebral contusion in left frontal lobe. The literature review revealed only a handful of reports of SSPE in toddlers [10–15] with a majority being reported to be a result of congenital measles infection [9–12]. Shorter latency period between measles virus infection and onset of symptoms of SSPE in absence of congenital measles infection has been reported only recently. The exact cause for this changing epidemiological trend remains to be ascertained. With this changing epidemiological trend in SSPE, a high index of suspicion is needed to detect SSPE with atypical presentation like very early onset (toddler age group or maybe even infancy) and shorter latency period between history of postnatally acquired measles infection and symptom onset.
